# Metal(loid) speciation and transformation by aerobic methanotrophs

**DOI:** 10.1186/s40168-021-01112-y

**Published:** 2021-07-06

**Authors:** Obulisamy Parthiba Karthikeyan, Thomas J. Smith, Shamsudeen Umar Dandare, Kamaludeen Sara Parwin, Heetasmin Singh, Hui Xin Loh, Mark R Cunningham, Paul Nicholas Williams, Tim Nichol, Avudainayagam Subramanian, Kumarasamy Ramasamy, Deepak Kumaresan

**Affiliations:** 1 School of Biological Sciences & Institute for Global Food Security, Queen’s University Belfast, 19 Chlorine Gardens, Belfast, UK; 2 Civil and Environmental Engineering, University of Michigan, Ann Arbor, MI USA; 3 Department of Engineering Technology, College of Technology, University of Houston, Houston, TX USA; 4 Biomolecular Sciences Research Centre, Sheffield Hallam University, Sheffield, UK; 5 Department of Environmental Sciences, Tamil Nadu Agricultural University, Coimbatore, India; 6 Department of Chemistry, University of Guyana, Georgetown, Guyana; 7 Faculty of Science, SRM University, Chennai, India

**Keywords:** Methanotrophs, Metalloenzymes, Methanobactin, Metal transformation and speciation, Bioremediation

## Abstract

Manufacturing and resource industries are the key drivers for economic growth with a huge environmental cost (e.g. discharge of industrial effluents and post-mining substrates). Pollutants from waste streams, either organic or inorganic (e.g. heavy metals), are prone to interact with their physical environment that not only affects the ecosystem health but also the livelihood of local communities. Unlike organic pollutants, heavy metals or trace metals (e.g. chromium, mercury) are non-biodegradable, bioaccumulate through food-web interactions and are likely to have a long-term impact on ecosystem health. Microorganisms provide varied ecosystem services including climate regulation, purification of groundwater, rehabilitation of contaminated sites by detoxifying pollutants. Recent studies have highlighted the potential of methanotrophs, a group of bacteria that can use methane as a sole carbon and energy source, to transform toxic metal (loids) such as chromium, mercury and selenium. In this review, we synthesise recent advances in the role of essential metals (e.g. copper) for methanotroph activity, uptake mechanisms alongside their potential to transform toxic heavy metal (loids). Case studies are presented on chromium, selenium and mercury pollution from the tanneries, coal burning and artisanal gold mining, respectively, which are particular problems in the developing economy that we propose may be suitable for remediation by methanotrophs.

Video Abstract

**Supplementary Information:**

The online version contains supplementary material available at 10.1186/s40168-021-01112-y.

## Introduction

The world’s population is predicted to reach 9.7 billion by 2050. Increasing demand for food and energy contributes to over-exploitation of natural resources and environmental degradation. Particularly, release of pollutants into the environment from manufacturing and resource industries is a major concern for ecosystem health. Pollutants, either organic (e.g. polycyclic aromatic hydrocarbons) or inorganic (e.g. heavy metals), when they interact with their physical environment, not only affect the quality of the environment but also have cascading effects on the health and wellbeing of organisms across all domains of life [1, 2]. Unlike organic pollutants, heavy metal(loid)s or trace metals (e.g. chromium-Cr, mercury-Hg, selenium-Se) are non-biodegradable, persist longer in the environment and bioaccumulate through the food web. Even at low concentrations, they are likely to have a long-term impact on ecosystem health [3, 4]. Naturally occurring metals are integral to the evolution of living organisms and are critical for metabolic activities e.g. co-factors for enzymes [5]. Despite their biological importance, large amounts of these metals can result in cellular and tissue damage, i.e. cytotoxicity in animals and growth inhibition in microbes; poor growth, low yields and nutrient inbalances in plants and metabolic interferences and mutangenesis in all types of organisms [6, 7]. Detailed reviews on the occurrence of heavy metals in the environment, industrial production and usage, potential for human exposure, and their molecular mechanisms of toxicity can be found in [8–11]. In this review, we also highlight the impact of tannery industries (i.e. Cr pollution – Table 1), selenium-polluting industries including mining (Table 2), and artisinal gold mining (i.e. Hg pollution – Table 3) as case studies.

**Table 1:** Chromium pollution from tannery industries—a case study

Tannery industries contribute significiantly to the developing economies such as India and Bangladesh (~3.5 and 5 billion USD per annum, respectively). Leather production utilises a large amount of water. It has been estimated that about 25–40m^3^ of fresh/ground water resources is used and subsequently discharged into the environment as effluent during the processing of one tonne of hides. Tannery effluents generally contain high levels of organics (measured as biological/chemical oxygen demand), nitrogen, sulphate and heavy metals such as Cr, Ni, As and Co. Tanneries have been the subject of wide public debate, particularly the downstream pollution by carcinogenic and teratogenic Cr (VI) that leaches into water bodies and soil and its subsequent impact on ecosystem health. For example, the Vellore district in South India is a well-known tannery hub that is famous for its export of leather [12]. Extensive surveys on tannery-associated groundwater contamination have revealed that toxic Cr (VI) can be detected (even at a depth of 10 m) at a high concentration up to 38 mg L^-1^ (critical limit 0.05 mg L^-1^ [13];) in the Ranipet, Walajapet and Vaniambadi areas of the Vellore district. This is extremely high compared to levels reported in other parts of India (4–7 mg L^-1^) [14].	
Chromium pollution from tanneries extends to soil e.g. about 50,000 ha of agricultural land has been affected due to salts and chromium from the tannery waste streams. Concentrations of exchangeable Cr fractions have been reported up to 128 μg kg^-1^. Research in sites dumped with tannery wastes over the past 20 years in Vellore and surrounding regions has indicated that soil alkalinity facilitates the presence of the more toxic and mobile Cr (VI) that subsequently leaches into the groundwater. Alarming levels of Cr were also found in borewell waters in Palar river basin (>500 μg Cr L^-1^), 90% of which was Cr(VI) [15]. In highly contaminated zones, the total Cr was reported to be as high as 102 g Cr kg^-1^ soil and has been found even at soil depth of 30 cm (1.1 mg Cr(VI) kg^-1^ [16, 17]. While tanneries use Cr(III) salts for leather processing, the presence of Cr(VI) in the contaminated sites is still an intriguing question. Contrary to the general acceptance that the presence of organic matter and other species contributing to electron transfer reactions in soil would rapidly convert Cr(VI) to Cr(III), these soils showed higher levels of Cr(VI) despite high-soil organic content (15%) [16]. It has also been reported that high concentrations of sodium and phosphates in soil solution can also trigger Cr (VI) mobility in soils with alkaline pH [18]. In addition manganese oxides are reported to reoxidise Cr(III) to Cr(VI) [19]. While tanners are replacing tannins instead of chromium, remediation of Cr(VI) in long-term contaminated soils have not been successful owing to reoxidation of Cr(III) to Cr(VI) [20] and continue to be an major issue.

**Table 2:** Selenium an essential element with toxicity problems in the mining industry and beyond

The Recommended Daily Intake of selenium in the human diet is 55 mg d^-1^ (dietary reference intakes, 2000; Dietary Reference Intakes (2000) National Research Council. Washington: National Academic Press). The World Health Organization (WHO) has indicated that Se intake in the human diet in excess of 400 mg d^-1^ may be harmful to health, with signs of Se overexposure being evident at 750–858 mg d^-1^ [21]. Potentially, toxic levels of selenium in the environment may occur naturally due to the presence of seleniferous rocks and also due to human activities, particularly mining. Selenium concentrations in agricultural drainage water in the range 0.14–1.4 mg L^-1^ were reported to cause death and deformity in aquatic birds [22]. The WHO has set the maximum permitted Se concentration for drinking water at 40 mg L^-1^, although specific jurisdictions have set limits as low as 10 mg L^-1^. Water quality guidelines for freshwater and water used for agricultural irrigation water range from 1 to 150 mg L^-1^ [23]. Selenium is strongly enriched in coal compared to other rocks and so coal and the ash from coal combustion are major sources of toxic amounts of selenium. Selenium species enter the air due to combustion of coal. The selenium that remains in coal ash is predominantly in the toxic and water soluble selenite form. It is subject to sorption to various components of ash, though is generally mobile into the aqueous phase at acidic pH [24]. Waste water from coal mining operations may contain more than 1 mg L^-1^ of selenium [23]. Problems with Se (and other pollutants due to processing and burning of coal) are a particular concern in China, where coal production and use have more than doubled since 2000 and are predicted to continue to rise, while they have been stable in most other areas of the world [25].	
Other emerging industries may provide new sources of potentially harmful selenium exposure. Selenium is a significant element in waste electronic and electrical equipment (e-waste). One study in West Africa (Ghana) found a doubling in blood selenium concentration (together with a tripling of mercury levels) in workers involved in incineration of e-waste [26].	
As detailed in the main text, methanotrophs and other environmental bacteria have the capacity to produce Se (0)-containing nanoparticles. In addition to being valuable in detoxifying selenium contamination and in providing novel nanoparticles for use in electronics, such nanoparticles may find uses as slow-releasing selenium supplements for diets [27].

**Table 3:** Mercury pollution from artisanal/small scale gold mining

Mercury emissions from artisanal and small scale gold mining, estimated at 727 tonnes per annum, account for a large portion of emissions from anthropogenic sources (37% [28];). Telmer and colleagues also estimated the contribution of artisanal and small scale gold mining to mercury releases between 640 and 1350 tonnes per year from at least 70 countries, with at least 350 tonnes emitted directly into the atmosphere while the remainder are released into the rivers, lakes, soil and tailings [29]. In small developing countries such as Guyana (Fig. 4), the gold mining industry is economically significant, where it contributed 13.7% to the total GDP of the country and accounted for more than 60% of total exports (USD 817.5 million) in 2017 [30]. The artisanal, small and medium scale operators, who contribute approximately two thirds of total gold declarations, rely almost exclusively on the use of mercury for gold extraction and concentration while the large scale companies utilise higher-recovering technologies with more control over environmental and safety risks [31]. Mercury is often added to the collected unprocessed gold ore in order to create a mercury-gold amalgam which is then heated to release the mercury and recapture the gold in concentrate. Most of the mercury vapour generated during burning of the amalgam may be collected by a retort, thus reducing mercury emissions by over 93%. However, studies in Guyana and Suriname have shown that while miners have some knowledge of the negative health and environmental effects of burning amalgams in the open air, they do not regularly use retorts for a variety of reasons, with the most common cited as the retorts being ‘too time-consuming’ [32, 33].	
In addition, mercury is sometimes used in sluice boxes and in panning which can also contaminate tailings, creeks and rivers which will leach into the surrounding environment. In the Minamata Initial Assessment conducted for Guyana, over 11,000 kg of mercury is estimated to be emitted annually in Guyana by burning of a mercury-gold amalgam, with 39% released in the air, 32% in water and 29% in land [33]. Mercury emitted to the atmosphere can be deposited into aqueous environments by wet and dry depositions, and some can be re-emitted into the atmosphere. In surveys carried out by the Guyana Geology and Mines Commission (2000 and 2001) in three rivers within two different mining regions of Guyana, it was found that 57%, 39% and 25% of predatory fishes sampled had mercury levels above the maximum World Health Organization guideline concentration (0.5 μg/g). Data from neighbouring Suriname and French Guiana, where mercury use in mining is also abundant, also indicate high levels of mercury contamination in fish [34]. In a study by Howard and colleagues, sediments taken from active and historically mined areas in Guyana had a mean mercury concentration of 0.229 μg/g, with a range from 0.029 to 1.2 μg/g, which is above Canadian Environmental quality guidelines (0.19 μg/g) [35]. There is also a lack of extensive data on mercury contamination in communities surrounding mining activities in Guyana. A study conducted from 2008 to 2010 by Singh and colleagues reported mercury concentrations of up to 70.8 μg/g (well over the WHO safe limit of 10 μg/g) in the hair of pregnant and nursing women from indigenous populations living close to small scale gold mining activities [36].	
Mercury has a long history of uncontrolled use in the mining sector of Guyana resulting in significant environmental pollution of waterways and aquatic ecosystems. The Government of Guyana has, however, signed the Minamata Convention and has subsequently aimed to phase out the use of mercury by 2022, with particular attention to the gold mining sector as part of this commitment. However, it has witnessed resistance by small miners who have not been able to adapt to other techniques as there is general lack of awareness and understanding of these technologies, along with a lack of fiscal incentives and barriers to accessing finance to transition from this cheaper alternative [31, 33, 37].	

Current remediation strategies of contaminated sites include chemical extraction (with acids or chelating agents), immobilisation, encapsulation and electrolysis. Although these strategies have been useful, they come with several limitations including significant alterations of the physicochemical properties, low efficiency and high cost of operation [38]. Phyto/bioremediation has been suggested as an alternative eco-friendly approach to detoxify metals from contaminated sites [39]. This approach leverages intrinsic biological mechanisms of plants and microorganisms to transform and/or bioaccumulate metals from the environment [1]. In particular, microorganisms possess remarkable abilities to bioaccumulate, retain and transform heavy metal ions [40–42] by taking advantage of reduction/oxidation (redox) and other processes e.g. modulating solubility of metals without changing the oxidation state of the metal [43, 44].

Metal(loids) exhibit different physical and chemical forms (i.e. differences in speciation) in the environment. Electronic configuration, oxidation state and ionic radius all define the chemical speciation of a particular metal and its fractionation (i.e. whether it is labile/inert, ligand complexed, precipitated or existing as a free ion). Consequently, the chemical form of a metal strongly influences its reactivity, toxicity, mobility and interaction with microorganisms in the environment [44, 45]. For instance, copper (Cu) becomes potentially toxic when it transitions between Cu(II) and Cu(I), soluble and toxic chromium (Cr(VI)) are less toxic when reduced to Cr(III), mercury (Hg(II)) becomes neurotoxic when its methylated (CH_3_Hg) and As(III) is more toxic than As(V) [46–48].

Recent research has highlighted the ability of aerobic methanotrophs, a specialised group of bacteria that can use methane (CH_4_) as a sole carbon and energy source, to transform metals (and also metalloids) such as Cu, Cr, Se and Hg [41, 49, 50]. Methanotrophs belong to the phyla *Proteobacteria* (classes *Alphaproteobacteria* and *Gammaproteobacteria),* Candidate division NC10 and *Verrucomicrobia*. Till date, more than 29 methanotroph genera and 8 families (i.e. *Methylococcaceae, Methylothermaceae, Crenotrichaceae, Methylocystaceae, Beijerinkiaceae, and Methylacidiphilaceae* and two currently unclassified families) have been identified within these phyla. Complete genome sequences for representatives of >23 genera are available in public repositories [51, 52]. Proteobacterial methanotrophs are active primarily in methane-oxygen counter gradients of oxic-anoxic interfaces [and in upland soils (high affinity atmospheric methane oxidisers)], while methanotrophs found in extremely acidic geothermal sites belong to the phylum *Verrucomicrobia*. Anaerobic microbial methane oxidation has been recently discovered that use reverse methanogenesis process to convert CH_4_ into CO_2_ [53, 54]. In contrast, the members of the candidate phylum NC10, such as *Candidatus* Methylomirabilis oxyfera, use all aerobic methane oxidated pathway-specific proteins, while it acquires oxygen through reduction of nitrite to the oxidation of methane via a unique oxygen-producing pathway [55]. The activity and diversity of methanotrophs and their impact on methane fluxes in different environments (e.g. landfills, rice paddies, natural gas seeps, hypogenic caves, saline lakes) have been studied extensively using both cultivation-dependent and molecular ecology tools [56–59]. In this review, we focus on recent developments in the physiology of aerobic methanotrophs with emphasis on their role in transformation and speciation of metals and metalloids such as Cu, Cr, Se and Hg. Detailed descriptions of the biochemistry and physiology of methanotrophs/methylotrophs are beyond the scope of this review and the reader is referred to [5, 60].

### Aerobic methane oxidation and metalloenzymes

**Fig. 1:**
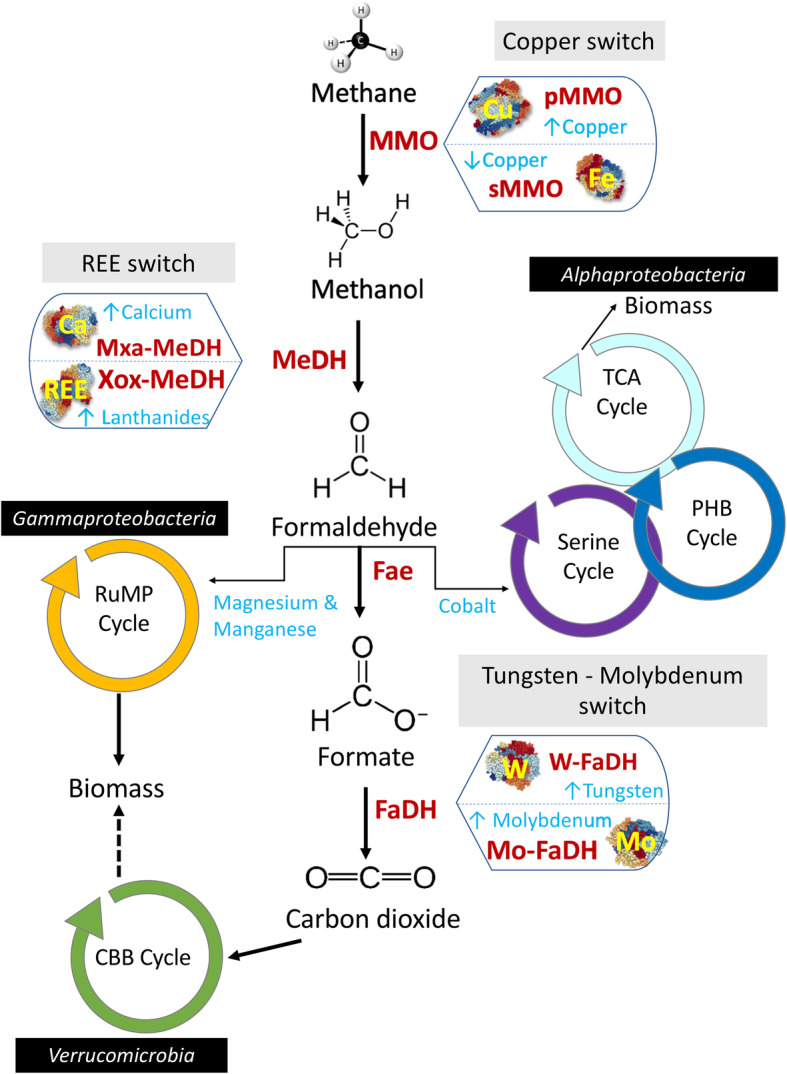
Methane oxidation by aerobic methanotrophs and metal co-factors of the enzymes. pMMO = particulate methane monooxygenase. sMMO = soluble methane monooxygenase. Xox-MeDH = XoxF-methanol dehydrogenase. Mxa-MeDH = MxaFI-methanol dehydrogenase. Fae = formaldehyde activating enzyme. FaDH = formate dehydrogenase. CBB = Calvin Benson Bassham Cycle. RuMP = Ribulose MonoPhosphate cycle. TCA – The Citric Acid cycle. PHB – Polyhydroxybutyrate cycle. Enzymes modulating the reaction are represented in red font, metals in blue & yellow fonts. Small vertical light blue arrows next to each metal ion indicate their effect on the expression and/or activity of enzymes

In aerobic methanotrophs, four major steps are involved in the enzymatic conversion of CH_4_ into biomass/CO_2_, in which the availabilities of different metal ions play a critical role (Fig. 1). The biocatalytic oxidation of CH_4_ to methanol (CH_3_OH) is modulated by the enzyme methane monooxygenase (MMO). Two forms of MMO exist: membrane-bound particulate MMO (known as pMMO and its divergent form pXMO [61]) and cytoplasmic soluble MMO (sMMO).

- ***pMMO*** composed (i) β-subunit, PmoA (26 kDa); (ii) α-subunit, PmoB (45 kDa); and (iii) γ-subunit, PmoC (23 kDa) with an (αβγ)_3_ structure. Their genes are typically arranged in pmo-operons as *pmoCAB*. Th general hypothesis is that the enzyme pMMO obtains electrons from the cytochrome *bc1* complex, which contains heme groups and Fe_2_S_2_ clusters [62, 63]. It has been proposed that Fe is required for pMMO activity [63], although a recent study shows only the presence of two mono-copper sites [64]. However, the alphaproteobacterial pMMO acquires electrons from ubiquinone pool through NADH oxidation [65, 66], while the pMMO activity in gammaproteobacterial methanotrophs is reported to be coupled to the oxidation of methanol to formaldehyde [67].
- ***sMMO*** is a well-characterised three-component enzyme: (i) hydroxylase contains 3 sub-units (α:β:γ – 54: 42: 22 kDa, respectively) with an (αβγ)_2_ structure; (ii) reductase (38–40 kDa) that supplies electrons from NADH to the hydroxylase; and (iii) component B as a regulatory protein (15–17 kDa) [68–70]. The sMMO is encoded by *mmoXYBZDC*. It contains a di-iron active site cluster in the hydroxylase component as well as an Fe_2_S_2_ cluster and flavin adenine dinucleotide (FAD) moiety in its reductase component [52].

The two types of MMO differ completely in their metal ion requirements, substrate specificity and CH_4_ oxidation kinetics [52, 64, 70–72]. Specifically, Cu(II) to biomass ratio determines the expression and activity of MMOs in methanotrophs that can express either form of the enzyme and is often referred to as the ‘copper switch’ [52, 61, 72–74]. Most aerobic methanotrophs possess pMMO or pXMO (with exceptions of alphaproteobacterial members within the genera *Methylocella, Methyloceanibacter* and *Methyloferula*), while few possess both pMMO and sMMO (e.g. *Methylosinus* spp.*, Methylocystis* spp.*, Methylobacter marinus, Methylocaldum marinum, Methylococcus capsulatus*, *Methylomagnum ishizawai* 175, *Methylomicrobium buryatense* 5G, *Methylovulum miyakonense* HT12, *Methylomonas* spp. [52, 75–77];). A very few methanotrophs e.g. *Methylocella* spp., *Methyloferulla* spp., *Methyloceanibacter* spp., (*Alphaproteobacteria*) and *Methyloterricola* spp. (*Gammaproteobacteria*) contain only sMMO (Vekeman et al., 2016; Semrau et al., 2018). The methane turnover number per active site cells producing pMMOs (0.5–2.5 s^-1^) are comparatively lower than sMMO expressing cells (>3.5 s^-1^) [70].

In the second step, methanol is converted into formaldehyde by the enzyme methanol dehydrogenase (MeDH), which contains a pyrroloquinoline quinone (PQQ) cofactor. There are two distinct types of MeDH, (a) calcium dependent MeDH (i.e. MxaF-MeDH) or (b) the recently discovered XoxF, which is an MeDH dependent on rare earth elements (REEs) e.g. cerium or lanthanum [5, 78–82].

- ***MxaF-MeDH*** is tetrameric (α_2_ – 66 kDa; β_2_ – 8.5 kDa), with greatest activity at pH~9 and requiring activation (e.g. by ammonia [83, 84];). The α sub-unit contains a single Ca^2+^ ion, the coordination sphere of which contains one nitrogen and two oxygen ligands from PQQ and a further four oxygen ligands from three aminoacyl sidechains (Glu 205, Asn 289 and Asp 331) [85].
- ***XoxF-MeDH*** is a α2 homodimer, located in the periplasm and active at neutral pH (optimum ~7). It coordinates the REE at the active site in a 9-coordinate fashion, via the same three ligands from PQQ and six oxygen ligands derived from four amino acyl residues (Glu 197, Asn 285, Asp 327 and Asp 329) of the protein [86]. It has been reported that the activity of purified XoxF was higher with light REEs (atomic numbers 57–63; La-Eu) in comparison to heavy REEs (atomic numbers 64–71; Gb-Lu) [87].

It should be noted that XoxF-MeDHs are also present in yeast, moulds, fungi and non-methylotrophic bacteria [88]. In methanotrophs such as *Methylomagnum ishizawai* 175, *Methylomicrobium kenyense* AMO1, *Methyloterricola oryzae* 73a, *Methylocystis* sp.*,* Mit Z-2018, *Methylosinus* spp*.,* R-45379, *Methyloacidiphilum fumariolicum* SolV and *Verrucomicrobium* spp., the only form of MeDH that have been identified is the XoxF-MeDH. The physiology and genetic basis of the ‘REE-switch’ that controls expression of the alternate forms of MeDH is reviewed extensively in [5, 52]. XoxF-MeDH is reported to have higher affinity for methanol and faster conversion rate [89]. While there was an assumption that XoxF-MeDH oxidises methanol to formate by dual activity [90, 91], a recent study by Good and Colleagues [92], using XoxF purified from *Methylobacterium extorquens* AM1, confirmed that formaldehyde is the final product. There are 5 families of XoxF-MeDH that might display different catalytic properties [93], while all need to be validated to conclude whether the dual activity is relevant in vivo or not.

In subsequent steps, the formaldehyde derived from the oxidation of methanol is assimilated as biomass either via the ribulose monophosphate pathway (RuMP) in most of *Gammaproteobacteria* or the serine pathway in *Alphaproteobacteria*. There are multiple pathways for oxidation of formaldehyde to CO_2_ i.e. in conjugation with tetrahydromethanopterin (H_4_MPT) or tetrahydrofolate (H_4_F) [94].

- **The multi-step H**_**4**_**MPT- and H**_**4**_**F-dependent pathways**, which also exist in non-methanotrophic methylotrophs such as *Methylobacterium extorquens*, operate by conjugating the formaldehyde and subsequent intermediates with the respective coenzymes. In model methanotrophs such as *Mc. capsulatus* (Bath) and *Ms. trichosporium,* OB3b studies have shown the use of both H_4_MPT- and H_4_F-dependent pathways [95–98].
- ***DL-FalDH*** is homotetramer with a sub-unit mass of ~ 49 kDa, while the sub-unit contains a PQQ as red-ox co-factor at 1:1 ratio. It utilises cytochrome *b*_559/569_ complex as a electron acceptor [99].
- ***N-FalDH*** was reported as an NAD(P)^+^-linked dehydrogenase from *Mc. capsulatus* (Bath) where the ability to oxidise formaldehye depended on a low molecular-mass heat-stable component [100, 101]. Later attempts to replicate these results yielded preparations that were active but found to be mixtures of other enzymes and cofactors involved elsewhere in the methane oxidation pathway [102]. Hence, whether an enzyme that directly catalyses NAD(P)^+^-linked oxidation of formaldhyde is a substantial contributor to the aerobic methane oxidation pathway remains unclear.

However, cytochrome *bc1* (with Fe) is required for FalDH as an electron acceptor. Also, other enzymes involved in the conversion of methanol into formaldehyde, formate and finally into carbon dioxide primarily rely on Fe, with minor requirements of Cu, Ca, Mo and Zinc (Zn) ions. In particular, the membrane-associated formate dehydrogenase (FaDH) enzyme, which is 2 αβγδ protomers (Mw ~400 kDa). The holoenzyme contains flavin, iron, inorganic sulphide and molybdenum [103]. The FaDH is involved in the conversion of formate to CO_2_, contains four Fe_x_S_x_ clusters and molybdenum (Mo) as cofactors [62]. Recently, the presence of two clusters encoding NAD^+^-dependent FaDH that require either tungsten (W) or Mo was reported [90]. Moreover, it was also observed that in *Methylomicrobium alcaliphilum* 20ZR formate production was considerably reduced during the presence of W [90]. Further work is required to develop a better understanding of whether a ‘W-Mo switch’ regulates expression of the two types of FaDH.

### Metal uptake mechanisms in aerobic methanotrophs

Metal ions are integral to metabolic activities (Table 4) in methanotrophs. It has been estimated that about one quarter to one third of any bacterial cell proteins required metal ions to support their functions [104]. However, cells can restrict the number of metal ions to be transported into their cytoplasm, thereby creating a competition between different proteins requiring the same metal ions and thus influencing different enzyme activities in vivo. Specific energised pumps are used to facilitate metal ion transport either into the cell (importers) or out of it (exporters). Such transporters are generally located in the cell membrane and may use selective metal binding proteins or small molecules such as siderophores [105]. In methanotrophs, metals are transported into the cell by (a) passive diffusion e.g. direct transport through porins in surface membrane and/or (b) active transport e.g. through TonB-dependent metal transport or other metal transport proteins [106, 107].

**Table 4:** Redox states of metals with metallo-enzymes and their specific catalytic functions

Metal	Redox state	Enzymes	Class of catalysis by enzyme
Copper	Cu (II), Cu (I)	Most copper-containing enzymes (e.g. Cytochromes)	Electron transfer, ferrous oxidase, amine oxidase
Iron	Fe (II), Fe (III), Fe (IV), Fe (V)	Cytochromes Peroxidase, catalase	Electron transfer, Oxidation
Molybdenum	Mo (III) to Mo (VI)	Nitrogenase, Aldehyde oxidase	Oxidation
Cobalt	Co (I)?, Co (II), Co (III)	B12- requiring enzymes	Carbonic anhydrase
Manganese	Mn (III) to Mn (IV)?	Photosynthetic enzymes	Superoxide dismutase, oxidase
Chromium	Cr (VI) to Cr (III)	Dehydrogenase	Oxidoreductases

#### Passive diffusion of metals

Passive transport of metals and organic solutes usually occurs via porins. The pore size and amino-acid compositions in the channel of porins determine their specificity towards different solutes and transport. In bacteria, the performance of porins (both non-specific and specific diffusion channels) is usually regulated by the availability of specific nutrients and downregulated by the presence of toxins or harmful solutes [108]. Based on their structure and diffusion characteristics, porins are classified as (a) specific monomeric, (b) specific trimeric, (c) non-specific monomeric and (d) non-specific trimeric diffusion channels [109], while little is known about the porin structures in methanotrophs. Reseachers have characterised a small number of outer membrane proteins (e.g. MopE and CorA) that can perform the function of copper transport in methanotrophs [106]. While MopE (some methanotrophs from *Gammaproteobacteria* express it) has been reported to bind copper with high affinity (< 10^20^ M^-1^) and to have a binding site composed of two imidazoles and a kynurenine (modified tyrptophan side-chain) group [110], other outer membrane proteins, as wells as their arrangements are yet to be characterised in detail. Unchelated metal ions are also reported to be transported via porins e.g. Cu and Hg transport in *M. album* BG8 [50].

#### Active transport of metals

It has been reported that about 10^−6^ M of intracellular ion concentration is required for any cellular activity and at lower levels, metal binding proteins or small molecules (e.g. siderophores) are synthesised by bacteria (including methanotrophs) to scavenge metals from the environment. For example, methanotrophs produce methanobactin under Cu limited conditions [111, 112]. Methanobactin, a chalkophore (chalk – copper in Greek), is a ribosomally produced and post-translationally modified peptide [72, 111, 113]. It plays a key role in active transport of metals and perhaps also enables ecological succession of different methanotrophs under external metal toxicities [72]. Specifically, the methanotrophs can export up to 3–50 methanobactin molecules per cell per second dependent on the Cu concentrations in the external solute [72]. Only very few methanotrophic species from the *Alphaproteobacteria* family are reported to biosynthesise methanobactin and only 10% of sequenced methanotrophs contain methanobactin biosynthesis genes [72, 74, 114]. Methanobactins produced by different methanotrophs are structurally distinct (i.e. till date only 7 methanobactins are characterised from methanotrophs), though with similar metal (i.e. Cu) binding sites, and are categorised as Group-I and II (Table 5). *Ms. trichosporium* OB3b was reported to produce the highest concentrations of Group-I methanobactin (35–60 mg l^−1^) followed by the *Mc. capsulatus* (Bath) (18–24 mg l^−1^) [115]. Interestingly, *Mc. capsulatus* (Bath) does not have the *mbn* cluster encoding methanobactin production [116, 117] indicating a different type of copper-binding molecule (including but possibly not restricted to MopE [118] used in high-affinity copper acquisition). Group-II methanobactin is mainly produced by *Methylocystis* sp. strain SB2, while recent bioinformatic analysis has shown that there are few methanotrophs that can make both the forms of methanobactins e.g. *Methylocystis parvus* OBBP*, Methylocystis* sp. LW5*, Methylosinus* sp. LW3*, Methylosinus* sp. R-45379 and *Methylosinus sav2* [112].

**Table 5:** Difference between two well characterised known groups of methanobactins

Particulars	Group 1 methanobactin (e.g. *Ms. trichosporium* OB3b)	Group 2 methanobactin (e.g. *Methylocystis* strain SB2)
Molecular weight (Da.)	1154.26	851.20
Structural difference	Two oxazolone rings (UV-vis spectra: Ring A ~ 394nm and B ~ 342nm)	One oxazolone ring (UV-vis spectra: Ring B~ 338nm) and Imidazolone (UV-vis spectra: Ring A ~ 387nm)
Partial amino-acids associated with Ring-A	Leucine	Arginine
Partial amino-acids associated with Ring-B	Proline	Threonine
Amino acids	Gly^1^, Ser^2^, Cys^3^, Tyr^4^, Ser^5^, Cys^6^ and Met^7^ (Gly^1^ – Downfield shift at 9.28ppm)	Ala^1^, Ser^2^, Ala^3^, Ala^4^ (Ala^1^ – Downfield shift at 11.7 and 145 ppm)
Copper affinity	10^18^–10^50^ M^-1^	10^26^ M^-1^
Copper binding Ring-A	640 S^-1^	Not available
Copper co-ordination rate with Ring-B	121 S^-1^	>2000 S^-1^
Structure modification	Pyramid-like structure	Hairpin-like structure
Stability	Both rings hydrolysed within 2–5 days under acidic condition	Ring B (i.e. Oxa) is susceptible to hydrolysis (200 min) and Ring-A more resistance
Disulphide bond	Found	Not found
Sulphate group	Not found	Found

*Note: Da.* daltons, *UV* ultraviolet, *Gly* glycine, *Ser* serine, *Cys* cystine, Tyr tyrosine, Met methionine, *ppm* parts per million

**Fig. 2:**
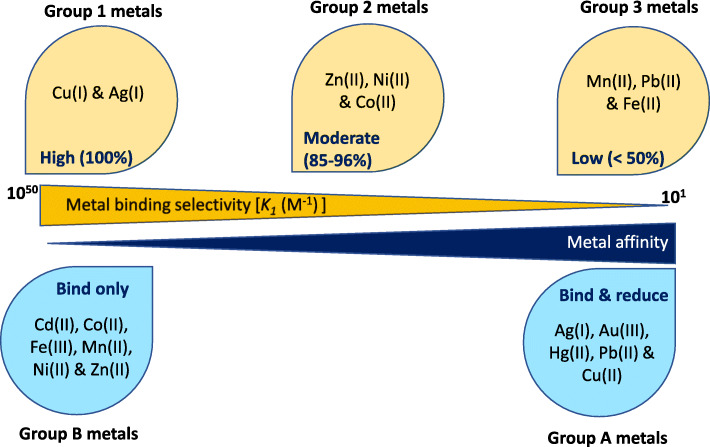
Grouping of metals based on their affinity and selectivity with methanobactin

As discussed earlier, methanobactin is reported to show high affinity towards Cu (~10^11^ to 10^34^ M^−1^) followed by Ag (~10^7^ M^−1^) as summarised in Fig. 2. Based on the metal binding properties of methanobactin, metals are grouped under two categories: Group A metals—Ag(I), Au(III), Hg(II), Cu(II) and Pb(II) and Group B metals—Cd(II), Co(II), Fe(III), Mn(II), Ni(II) and Zn(II) [118]**.** Group A metals bind at both oxazolone rings and are reduced upon binding, while Group B metals bind at just one oxazolone ring and are not reduced upon binding [118, 119]. Based on the methanobactic metal selectivity, the metals are grouped as (a) Group-1 (high—100%): Cu (I) and Ag (I); (b) Group 2 (moderate—85–96%): Zn (II), Ni (II) and Co (II); and (c) Group 3 (low—<50%): Mn (II), Pb (II) and Fe (II). Studies have also shown that the methanobactin can also bind, transform and detoxify Hg [50, 69, 120] and U (VI) [121]. In a recent study, a methanobactin characterised from *Methylosinus sporium* was found to have one oxazolone and one imidazolone ring with disulphide bond between two Cys residues as in Group 1 [116]. The structural difference may affect the metal binding and affinity, which is not completely understood yet. So the classification, metal affinity and grouping of metals described above may applicable only to the methanobactins from *Methylosinus trichosporium* OB3b and *Methylocystis* sp. strain SB2, while it may vary for other methanobactin structures and requires further investigation. This knowledge is critical in determining how to exploit methanotroph strains and/or methanobactins to sequester the metal ions from the environment.

### Copper accumulation by methanotrophs

Copper is central to methanotrophic activity, while 40% of methanotrophs are reported to have Cu storage proteins and only 10% reported to make methanobactins for Cu uptake, while the remainder may use passive transport of metals using other porin related proteins [122]. It has been estimated that methanotrophs have at least a ten-fold greater Cu requirement compared with other bacteria [123]. While Cu is an essential metal ion for pMMO activity (i.e. CH_4_ to methanol conversion), it has also been shown to control expression of other enzymes (e.g. formaldehyde dehydrogenases, hemerythrin) and outer membrane proteins involved in Cu assimilation, regulation and transport. Moreover, it can influence the membrane structure or formation in methanotrophs e.g. *M. capsulatus* (Bath), *M. album* BG8 and *Ms. trichosporium* OB3b [123, 124]. The Cu-containing protein pMMO, which constitutes up to 20% of the total cellular proteins in methanotrophs [125] shows high-affinity towards CH_4_. The pMMO is expressed at high Cu concentrations, and at low levels, sMMO is expressed [126]. At high Cu concentrations, the Cu will bind the active site of sMMO and inhibit the electron transfer between the falvin adenine di-nucleotide and MMOH [127]. High copper-to-biomass ratio leads to pMMO expression through the canocial ‘copper switch’, but the exact mechanisms by which Cu activates pMMO expression is not yet clear. However, the rate of copper uptake by passive diffusion or active transport may differ between methanotrophs, mainly in *Alphaproteobacteria* and *Gammaproteobacteria*. During active transport, divalent Cu is reduced to monovalent Cu, which forms a more stable complex with methanobactin that will not be disassociated by simple dissolution mechanisms and transport into the cell. The methanobactin-Cu (I) complexes (at 1:1 stochiometry [119];) are reported to be too large to be transported via porins, and their transport is mainly mediated by outer membrane TonB-dependent transporters (TBDTs [106, 107];). However, it should be noted that the mechanism of Cu reduction by methanobactin is still unknown [111], while Cu concentrations and pH are known to influence the metal-methanobactin interactions [115, 128]. The methanobactin-Cu complex is also reported to regulate reductase-dependent oxidase activity, dismutation of O_2_ to H_2_O_2_, and the reductant-dependent reduction of H_2_O_2_ to H_2_O [129]. Recently, researchers have characterised the novel Csp-proteins i.e. Csp1 and Csp2 that were reported to be a major reservoir for Cu (up to 13 Cu(I) in one Csp protein molecule; affinity ~ 10^17^ M^-1^) and proposed to be exported from the cytosol to supply copper to pMMOs [130]. The related protein, Csp3, can hold upto 80 Cu(I) ions. Csp3 is speculated to sequester excess copper in the cytosol and rescue cellular activity from metal toxicity. Csp3, which also occurs in non-methanotrophs including *Bacillus subtilis*, is the only bacterial system known to store Cu in the cytoplasm [122]. The mechanism of Cu(I) release from the metallo-proteins methanobactin and the Csp is still not clear. Considering their copper requirements and ability to produce Cu-binding proteins and peptides, methanotrophs could be potentially exploited for copper extraction and recovery from Cu ore, minerals and tailings or remediation of Cu contaminated sites using either whole cell or immobilised protein based approaches [131]. Existing bioleaching methods work effectively under acidic pH (~2.0), whereas methanotroph-based bioleaching could work effectively under neutral pH and thus be considered as more eco-friendly.

### Interaction of methanothrophs with chromium (VI)

Chromium is found in the environment mainly in its two most stable oxidation states, the highly soluble, bioavailable and oxidising hexavalent form and the less toxic, less soluble and less bioavailable trivalent form [132]. Despite world-wide regulation of the use of hexavalent chromium in metal plating, to inhibit corrosion and as a wood preservative, among other applications, hexavalent Cr (VI) continues to be a substantial environmental problem. Chromium is heavily used in a number of industries, especially in the production of chromium-iron alloys such as stainless steel and the use of Cr (III) salts in leather manufacture (see Table 1 for case study on the extent of Cr pollution from tannery industries). In 2017, total production of chromium-iron alloys was 31 million tons. In which, 70–80% comes from South Africa (15 MT), Kazakhstan (5.4 MT), India (3.2 MT) and Turkey (2.8 MT), while China and the USA are the top consumers of Cr alloys. Anthropogenic Cr contamination contains a significant amount of Cr (VI) which may leach into the aqueous environment [12, 133].

**Fig. 3:**
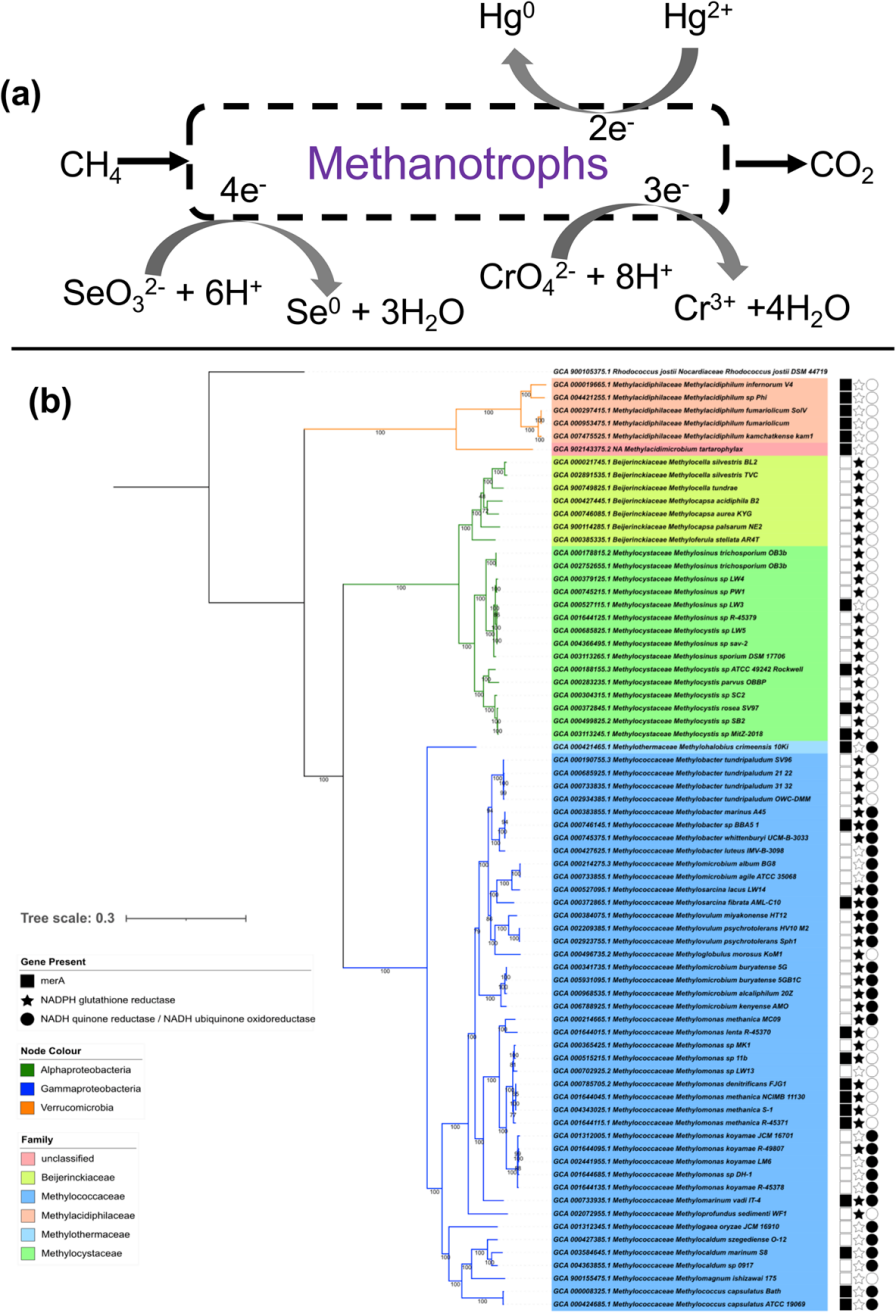
**a** Pathways of methane-driven metal biotransformation by obligate aerobic methanotrophs. **b** Genomic distribution of potential biomarker genes involved in metal transformation in methanotrophs. Presence/absence of biomarker genes are mapped to a phylogenomic tree constructed using 74 single-copy marker genes specific to *Bacteria* via the GtoTree (v1.5.22) pipeline (as described in [135]). Protein sequences were retrieved using HMMER3 tool and multiple alignments were produced using MUSCLE (v.3.8.31, default settings). Conserved alignment blocks were identified using trimal (v1.4; -automated1 option) and subsequently used for tree construction using the IQTREE2 (v2.0.3) using default setting and 1000 boostraps

A wide range of bacteria including methanotrophs have been found able to bioremediate Cr(VI) by reducing it to the less harmful trivalent form [134]. Among the well characterised methanotrophs, *Methylococcus capsulatus* (Bath) is able to reduce Cr(VI) over a wide range of concentrations (tested across 1.4 to 1000 mg L^-1^), which offers the attractive possibility of using cheaply available methane to drive bioremediation of Cr(VI) and to sequester the Cr(III) product in the insoluble fraction, associated with the bacterial biomass (Fig. 3a). X-ray spectroscopy has confirmed that the Cr is in the +3 oxidation state and appears to have oxygen and phosphorous ligation [41]. Cell fractionation, together with in situ analysis of chromium distribution via X-ray photoelectron spectroscopy (XPS) and transmission electron microscopy coupled to energy-dispersive X-ray spectroscopy (TEM-EDX) showed that the chromium is predominantly intracellular [136]. Cells of the alphaproteobacterial methanotroph *Ms. trichosporium* did not reduce chromium (VI), although another gammaproteobacterial methanotroph *Methylomonas koyamae* SHU1, has been identified as able to reduce chromium (VI) [137]. Bioinformatic analysis of the genome of *Mc. capsulatus* (Bath) revealed five candidate reductases homologous to enzymes from other microorganisms known to reduce Cr(VI) [41]. Analysis of representatives of all methanotroph genera for which genome sequences is available indicates the presence of multiple potential chromium (VI) reductases in all of them. Comparative genomics of the three species for which the chromium (VI) reduction phenotype is known shows that only one homologue, a putative Na^+^-translocating NADH-quinone reductase subunit F (locus tag MCA2384 in *Mc. capsulatus* (Bath)), correlates with the ability to reduce chromium (VI) and so is a candidate for the chromium (VI)-reducing activity. This homologue is not found in any currently available alphaproteobacterial genomes and is present in 29 out of 44 gammaproteobacterial methanotroph genomes (Fig. 3b).

A mixed culture in a membrane biofilm reactor system was found able to reduce chromium during continuous operation (feeding at 1–3 mg L^-1^ of Cr (VI)) with a microbial consortium that contained microorganisms of the genera *Meiothermus* and *Methylosinus*. It was concluded that the reduction of Cr (VI) was performed primarily by the *Meiothermus* utilising multicarbon nutrients released by the *Methylosinus* growing on methane [20]. This is consistent with observations that pure cultures of the well characterised alphaproteobacterial methanotroph *Ms. trichosporium* OB3b did not reduce Cr (VI) [41], while a mixed culture of *Ms. trichosporium* OB3b and a Cr (VI)-reducing strain of *Escherichia coli* was able to reduce Cr (VI) using methane as the only externally supplied carbon source (A. Al Hasin, T.J. Smith and P.H.E. Gardiner, unpublished observations). Collectively, these data indicate that methanotrophs, whether in pure culture or mixed microbial communities, have the capacity to bioremediate Cr (VI) contamination over a wide range of concentrations using cheaply available methane as the feedstock.

### Interaction of methanothrophs with selenium

Selenium is an essential micronutrient across all domains of life (including prokaryotes and humans), principally because of its role in selenocysteine in certain enzymes such as glycine reductase, formate dehydrogenase, glutathione peroxidase, iodothyronine deiodinase and thioredoxin reductase. Selenium is also a substantial environmental problem, where the toxic and water-soluble oxyanions selenite (SeO_3_^2-^) and selenate (SeO_4_^2-^) may be present from natural and anthropogenic sources and are a risk to humans, animals and other forms of life [138] (see Table 2 for case study on problems of selenium associated with the mining industry and beyond). Some bacteria are able to respire using selenate (SeO_4_^2-^) as their terminal electron acceptor, while others are able to reduce selenium species to elemental selenium and perform methylation reactions [40, 139]. The primary methylated forms of selenium produced by microorganisms are dimethyl selenide (CH_3_-Se-CH_3_) and dimethyl diselenide (CH_3_-Se-Se-CH_3_), although others including dimethyl selenone [(CH_3_)_2_SeO_2_], dimethyl triselenide (CH_3_-Se-Se-Se-CH_3_), methyl selenol (CH_3_-Se-H) and mixed selenium/sulphur-methylated species such as dimethyl selenyl sulphide (CH_3_-Se-S-CH_3_) and dimethyl selenyl disulphide (CH_3_-Se-S-S-CH_3_) have also been observed [40, 49]. Elemental selenium is insoluble and so is generally considered the most benign form of the element with lowest bioavailability. When ingested by mice, Se as nanoparticulate Se(0) had 7-fold lower LD_50_ compared with Se as selenite [140]. In contrast, ingestion of Se(0) nanoparticles by fish has about 5-fold lower LD_50_ compared with selenite. A study using the estuarine invertebrate *Potamocorbula amurensis* suggested that Se(0)-rich particles produced by environmental consortia were more bioavailable than nanoparticulate Se(0) produced chemically or by pure bacterial cultures [141]. The diversity of selenium nanoparticles that can be produced by microorganisms may find applications in electronics and other industries [142–144].

Pure cultures of the *Mc. capsulatus* (Bath) or *Ms. trichosporium* OB3b do not detectably transform selenate, although both are able to remove selenite. The principal product is elemental selenium in the form of extracellular nanoparticles (Fig. 3a), as well as a small proportion that is converted to methylated Se species. The removal of selenite occurs more rapidly in *Mc. capsulatus* (Bath) compared with *Ms. trichosporium* OB3b (2-fold difference in rate observed in comparable tests in the laboratory), at the respective optimum temperatures (45 and 30°C) of the two strains. Cultures of *Mc. capsulatus* (Bath) completely removed selenite from a starting concentration of 40 mg L^-1^ within 50 h, with 75% conversion to elemental Se and the production of detectable methylated species (Fig. 3a [49];).

While it is not clear whether all volatile selenium species are produced via elemental Se reduction, *Mc. capsulatus* (Bath) and *Ms. trichosporium* OB3b are each able to transform selenium nanoparticles into methylated species. Various mixtures of Se volatiles were detected, depending on the methanotroph strain used and the type of Se supplied (selenite, biogenic selenium nanoparticles produced by the methanotroph, or chemically produced selenium). *Mc. capsulatus* supplied with selenite produced the largest number of detectable Se volatiles: dimethyl selenide (CH_3_-Se-CH_3_), dimethyl diselenide (CH_3_-Se-Se-CH_3_), dimethyl selenyl sulphide (CH_3_-Se-S-CH_3_), methyl selenol (CH_3_-Se-H) and methyl selenoacetate (CH_3_-Se-CH_2_-COOH [49];).

A study by Lai and colleagues indicated that methane-driven conversion of selenate to elemental Se is possible [142]. A mixed community of microorganisms operating under anoxic conditions was able to perform methane-dependent reduction of selenate to elemental selenium. This consortium contained a substantial population of genera associated with aerobic methane oxidation, particularly *Methylomonas.* A more recent study of a methane-driven selenate-reducing community in a membrane biofilm reactor, in which the predominant methanotrophs were *Methylocystis*, showed that the rate of selenate reduction peaked at an intermediate rate of oxygen supply. These results are consistent with a role of methane monooxygenase in the methanotrophs in oxygen-dependent conversion of methane to methanol as the principal carbon and energy source of the community. Cross-feeding from methanotrophs to non-methanotophs then enables reduction of selenate, via a reaction that is suppressed in the presence of excess oxygen. Consistent with this explanation, as the oxygen delivery rate was decreased to the intermediate level at which the rate of methane-driven selenate reduction was maximised, expression of pMMO genes (*pmoA*) decreased only 5.4-fold. At the same time, expression of nitrate reductase genes (*narG*, which may be involved in selenate reduction in non-methanotrophs including *Variovorax* and *Arthrobacter*) increased 50-fold [145].

The reduction and methylation of Se species by methanotrophs or consortia offer the possibility for methane-driven remediation and concentration of Se species, as well as producing Se as nanoparticles with novel properties that may be useful in electronics and other technologies, and for other uses such as addition as a micronutrient to selenium-poor foodstuffs.

The reduction of selenite to elemental selenium has been attributed to a range of reductase enzymes and also to non-enzymatic reactions of selenite with thiol-containing molecules such as glutathione [40]. In *Mc. capsulatus* (Bath), analysis of the low molecular weight selenium-containing compounds during transformation of selenite and Se(0) suggested an extracellular mechanism of selenite reduction with methyl selenol as an intermediate [146]. Glutathione reductase, which has been implicated in the reduction of selenite in microorganisms including *Pseudomonas stutzeri* [147], appears to be absent from the genome of *Mc. capsulatus* (Bath), though is distributed among alpha- and gamma-proteobacterial methanotroph genomes (Fig. 3b), being present in at least 49 out of 66 such genomes including that of *Ms. trichosporium* OB3b, the alphaproteobacterial methanotroph known to reduce selenite to Se (0).

### Mercury detoxification by methanotrophs

**Fig. 4:**
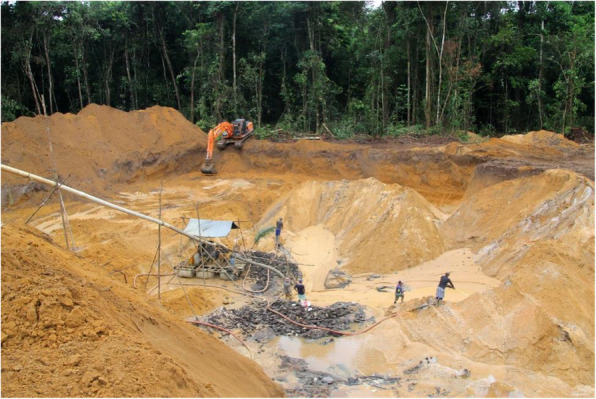
A typical artisanal/small scale gold mining operation in Guyana

Mercury (II) or Hg (II), a priority pollutant is released into environment to the tune of 4500–7500 tonnes per year. Around 55% (~1500 tonnes per annum) of global industrial mercury emissions arise from China, India and the USA [28]. Coal-fired industries, especially power generation, have been identified as the major source of mercury pollution alongside gold mining industries (Fig. 4; see Table 3 for case study on the extent of Hg pollution from artisanal/small scale gold mining from Guyana) [29, 148]. In the environment, elemental mercury (Hg(0)) is oxidised to inorganic Hg (II), which can then react with various organic compounds in water and soil sediment by biotic reactions facilitated by bacteria, and abiotic reactions mediated by sunlight photolysis, resulting in conversion into organic mercury such as methylmercury [148]. Methyl mercury (CH_3_Hg^+^), which is a more toxic form than Hg (II) or Hg (0), can easily be absorbed by organisms from the lower levels of the food chain and accumulated in higher trophic organisms e.g. through fishes to humans. Methyl mercury can affect the human body negatively, especially the nervous system and is particularly dangerous for pregnant women as the foetus can be affected by mercury passing through the placenta. Damage done to the brains of babies leads to symptoms such as deafness, blindness, microcephally, cerebral palsy and problems with swallowing [28]. Methanotrophs are able to reduce mercury and detoxify methyl mercury [149, 150]. Recently Shi and colleagues [150] reported the presence of all genes required for the reduction of Hg (II) in a metagenome-assembled genome of the alphaproteobacterial methanotroph *Methylocystis*. Our analysis of representative genomes (Fig. 3b) detected *merA* genes in most of the gammaproteobacterial

methanotrophs (13 out of 44), all of verrucomicrobial methanotrophs (6 out of 6) and in a very few alphaproteobacterial methanotrophs (4 out of 22). We also detected two copies of the *merA* gene in few methanotrophs such as *Methylacidiphilum fumariolicum*, *Methylacidiphilum kamchatkense* and *Methylocystis* sp. MitZ-2018. However, in other methanotroph genomes, we detected copies of *merA* homologues i.e. dihydrolipoamide dehydrogenase (DLD) and/or NADPH-glutathione reductase (*gorA*) genes. GorA, DLD and MerA belong to the family of flavin-dependent disulphide oxidoreductases that are mainly involved in redox reactions [151]. It has been reported that Hg (II) can bind strongly with sulfohydryl groups of proteins [152–155]. Previously characterised GorA and DLD enzymes are inhibited by Hg (II) upon binding at their sulfohydral binding sites, while MerA is catalytically active in the presence of Hg (II) [156]. This difference in the inhibitory effects of Hg (II) could be mainly due to the structural and redox cycling differences between the GorA or DLD and MerA [156, 157]. The possible role of DLD and GorA homologous proteins on Hg (II) or CH_3_Hg^+^ reduction and or detoxification in methanotrophs needs to be investigated.

It has been reported that there are two different mechanisms through which the specific enzymes are deactivated by binding of Hg (II). First mechanism: Hg (II) usually binds with proteins containing thiols (e.g. cysteine) and thioethers (e.g. methionine) causing protein unfolding, aggregation and precipitations [158]. Specifically, the N-termal cysteine residues are critical for Hg(II) binding and transport [156]. Second mechanism: In the absence of cysteine side chains, histidine binds Hg (II) at the constant of log *K*_f_-7.4 [159]. Moreover, Stratton and colleagues [158] reported that the S-ligands offer higher binding constants with Hg (II) (i.e. log *K*_f_ -14.4–52.7) in comparison to N-ligands (i.e. log *K*_f_ 3.5–8.8).

Methanobactin can bind Hg (II) similar to Cu (II) or Cu (I) (binding with both oxazolone rings at the rate > 2000 s^-1^) [52]. As discussed above, methanobactins contain cys-thiols groups that readily bind Hg (II). However, the binding ratios differ for methanobactins from *Ms. trichospoium* OB3b and *Methylocystis* sp. strain SB2, which might be linked to the presence of different heterocyclic rings and associated enithiols [119]. It has been reported that the binding of Hg:methanobactin ratio were different for *Ms. trichosporium* OB3b-methanobactin (i.e. 0.1) and *Methylocystis* sp. strain SB2-methanobactin (i.e. 0.5–6), while the Hg replaced the Cu from *Methylocystis* sp. strain SB2-methanobactin when added together. Moreover, the binding ratios of methanobactin with different Hg species (e.g. Hg(II), CH_3_Hg^+^ or Hg(CN)_2_) are significantly different for the two methanobactins due to their structural disparity.

Baral and colleagues [119] observed that Hg (II) is reduced (presumably to Hg (0) as inferred from the appearance of a grey colour) by methanobactin from *Ms. trichosporium* OB3b, but not from *Methylocystis* sp*.* strain SB2. Similarly, Hg (0) produced from Hg (II) by *Ms. trichosporium* OB3b producing methanobactin is not volatile but associated with the biomass at a protein:mercury mass ratio of approximately 2:1 [50]. Methanobactin has been shown to bind with CH_3_Hg^+^, particularly with oxazolone rings and not with other heterocyclic ring [119]. Methanotrophs lacking the ability to produce methanobactin are able to take up CH_3_Hg^+^ [42, 149], but their accumulation capacity is comparatively lower. It is hypothesised that the methanobactin facilitated uptake of CH_3_Hg^+^ (e.g. 100 nM to up to 500 μM) can result in conversion into inorganic Hg by an non-conventional ‘oxidative demethylation’ (i.e. not through the conventional organomercurial lyase encoded by *merB*) [52, 160]. In contrast to the CH_3_Hg^+^ degradation system of methanotrophs, MerB enzymes usually show very poor affinity for CH_3_Hg^+^ (Km around 500 μM) and are expressed only under certain environmental conditions such as concentrations of Hg > 1 μM and pH > 7.0 (optimum pH~ 10.2) [42]. Not all methanotrophs degrade CH_3_Hg^+^; e.g. *Ms. trichosporium* OB3b can degrade CH_3_Hg^+^, while *M. album* BG8 cannot. It has been speculated that the CH_3_Hg^+^ initially binds with methanobactin and is internalised for MeDH to cleave the C-Hg^+^ bonds in *Ms. trichosporium* OB3b, as shown by the inhibition of demethylation of CH_3_Hg^+^ when methanol is added. Overall, mechanistic understanding of demethylation characteristics in methanotrophs will allow researchers to develop remediation strategies for contaminated environments. While there is no evidence of Hg (II) methylation by methanotrophs, the role of methanobactin on Hg (II) methylation in the presence of *Geobacter sulfurreducens* PCA and *Desulfovibrio desulfuricans* ND132 was recently reported [120]. Since there are no studies reporting the specific binding proteins from methanotrophs other than methanobactin regulating Hg (II) and CH_3_Hg^+^ toxicity, there is a need to characterise methanotroph cell surface proteins with sulfohydral groups to understand their possible role in Hg (II) and CH_3_Hg^+^ binding and transport [155].

### Factors affecting metal transformation by methanotrophs

**Fig. 5:**
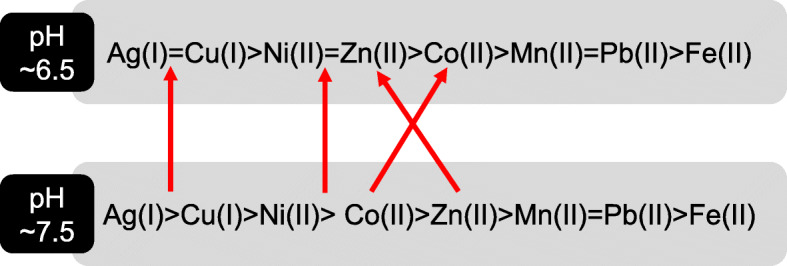
Order of metal affinity for methanobactin under different pH conditions (red arrow indicate the order of change in metal afficinity with respect to pH change)

A number of abiotic factors can affect the passive or active metal uptake systems and transformation by methanotrophs, among which pH, temperature, available oxygen concentrations, carbon source (e.g. CH_4_) and other metal(loid)s coordination play a critical role. Metal affinity of methanobactin is influenced by pH (Fig. 5 [119];) along with metal to methanotroph biomass (and methanobactin) ratio. The binding rate and affinity can alter with the molar ratio of metal to methanobactin (i.e. Cu and methanobactin). If the ratio is 0.5 or above, methanobactin binds as a monomer and at lower ratios as tetramer or oligomer. In the presence of other metals, Cu will be the preferred metal ion that will be readily taken up by the methanotrophs, while least preferred is iron. In presence of Ag (I) or Au (II), the copper uptake will be limited. Our understanding of the physiology of methanotrophs, particularly the regulation of genes (e.g. *TonB, mbnABCM, arsRBC, merABCD,* etc.) for metal uptake and/or transformations in relation to concentrations of Cu and other metals is limited. Uptake of Zn can be affected by Hg (II) or Cd, which are thiophilic in nature and bind to the cysteine in methanobactin. Temperature influences the solubility of methane in solution and thereby influences methanotrophic growth, which in turn may significantly influence metal ion uptake. However, there is no detailed understanding on how metal affinity for methanobactin and other methanotroph metal uptake systems varies at different temperatures. While methanotrophs with no capacity to make methanobactin can also uptake and accumulate metals, studies on these methanotrophs are also very limited. Pure cultures and mixed methanotrophs cultures with or without heterotrophs (that mimic natural conditions) may also behave differently in metal uptake and transformations and future research is required to understand metal transformations in near in situ conditions. Lai and colleagues [161] found that the available nitrate had significant impact on bromate reduction, while they also correlated it with polyhydroxyalkanoate (PHA) accumulation capacity. However, not all the strains can make PHA as storage material.

**Fig. 6:**
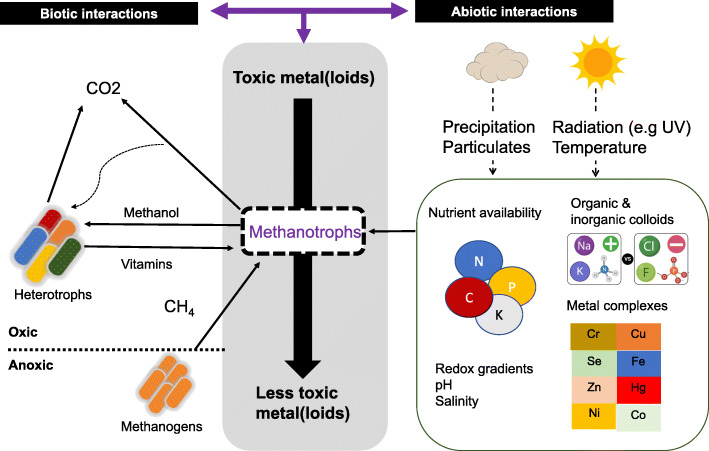
Schematic representation of potential biotic and abiotic interactions that constrains methanotroph-dependent metal(loid) transformation in the environment. N – nitrogen, P – phosphorus, K – potassium, C – carbon, Cu – copper, Fe – iron, Ni – nickel, Cr – chromium, Se – selenium, Hg – mercury, Zn – zinc, Co – Cobalt, Na – sodium, Cl – chlorine and F – flourine

A range of biotic interactions (e.g. methanotroph—heterotroph interactions; Fig. 6) can regulate methanotroph distribution, activity, metal uptake and transformation [71, 162, 163]. Both synergistic and antagonistic biotic interactions are known to impact methanotroph functional diversity and have been extensively reviewed in [162]. It has been well-established that exchange of metabolites between methanotrophs and heterotrophs improve methanotroph growth and activity [163, 164]. Methanotroph-heterotroph interactions are also constrainted by various abiotic factors. For instance, the ratio of CH_4_ to O_2_ altered the methanotroph-heterotroph community structure in the enrichment. In particular, PHB accumulating alphaproteobacterial methanotrophs dominanted with increasing CH_4_ content [165, 166]. Moreover, differences in Cu to Fe ratio were also found to impact community composition [167]. Our understanding on the role of biotic interactions on metal transformation by methanotrophs is currently limited. Given the potential role of methanotrophs in bioremediation strategies, there is an immediate need to explore how community level biotic interactions impact metal transformations and uptake.

## Conclusions and future considerations

Manufacturing and resources industries are key drivers for economic growth, yet this comes at a huge environmental cost affecting not only ecosystem services but also the livelihood of local communities. Aerobic methanotrophs are metabolically versatile and are able to detoxify toxic heavy metals such as chromium and mercury while growing on a cheap feedstock i.e. methane. In order to fully exploit these traits for mitigation of polluted sites, future research is required to better understand (i) physiological and genetic basis of Cr(VI) reduction and demethylation of mercury, (ii) mechanism of Cu reduction by methanobactin, (iii) role of porins in passive uptake of metals in methanotrophs, and (iv) role of molybdenum and tungsten in formate dehydrogenase activity. More importantly, our knowledge of metal transformations by methanotrophs is based to a large extent on laboratory strains. Further research is required to understand metal uptake/transformation mechanisms in the environment, particularly in polluted sites with elevated and/or multiple metal concentrations.

## Data Availability

The datasets (genome sequences) analysed during the current study are available through NCBI genome assembly repository (www.ncbi.nlm.nih.gov)
